# Comparative age distribution of influenza morbidity and mortality during seasonal influenza epidemics and the 2009 H1N1 pandemic

**DOI:** 10.1186/1471-2334-10-162

**Published:** 2010-06-09

**Authors:** Magali Lemaitre, Fabrice Carrat

**Affiliations:** 1 UPMC - Univ Paris 6, UMR-S 707, Paris, F-75012, France; 2 Inserm, UMR-S 707, Paris, F-75012, France; 3 Public Health Unit, Saint-Antoine Hospital, AP-HP, Paris, F-75012, France

## Abstract

**Background:**

Several studies have shown a relatively high mortality rate among young people infected by the 2009 pandemic influenza A (H1N1) virus. Here we compared the age distributions of morbidity and mortality during two seasonal influenza epidemics (H1N1 and H3N2) in France and the United States with those of the 2009 H1N1 pandemic waves in the same countries.

**Methods:**

Age-standardized ratios were used to compare the age distribution of morbidity and mortality due to influenza between the two countries and across the different years. Non parametric analysis of variance was used to compare these ratios between epidemic and pandemic influenza.

**Results:**

Age distribution of morbidity was similar between the 2009 pandemic and seasonal epidemics due to H1N1 (p = 0.72) and H3N2 viruses (p = 0.68). In contrast, the proportion of under-60s among influenza deaths was markedly higher during the 2009 pandemic (peak <20 years) than during the seasonal epidemics (respectively p = 0.007 and p = 0.0008).

**Conclusions:**

Young age was a principal mortality risk factor due to the 2009 H1N1 pandemic.

## Background

The 2009 pandemic influenza A (H1N1) virus (2009 pH1N1) emerged in April 2009 in the Americas and subsequently spread worldwide [1]. Preliminary findings in Mexico and the United States suggested that people aged from 5 to 30 years were at a higher risk of infection, while those aged 30 to 50 years old were at a higher risk of death [2-4]. The risk of infection and death seemed to be lower in the elderly population, a finding attributed to prior exposure to A/H1N1 viruses that circulated before 1957 [5,6]. During seasonal epidemics, mortality is classically highest in the elderly population. Conversely, during pandemic influenza mortality is highest in younger age groups. This shift of mortality toward younger age groups is considered as a signature feature of pandemics [7]. Few comparative studies are available on age distribution of morbidity during pandemic and seasonal influenza. Studies of the 1918, 1957 and 1968 pandemics suggest that the age distribution of morbidity was similar to that of seasonal influenza, but no direct comparisons are available [8,9]. Here we examined the age distribution of morbidity and mortality during seasonal influenza epidemics (H1N1 and H3N2) in the US and France by comparison with the 2009 H1N1 pandemic in the same countries.

## Methods

### Periods

We selected two seasonal influenza epidemics (H1N1 and H3N2) in the US and France, which were due to single viral strains and for which nationwide mortality and morbidity data were available.

The seasonal H1N1 epidemics were those of 1978-79 (A/USSR/90/77) in the US [8] and 1988-89 (A/SING/1/87) in France, while the seasonal H3N2 epidemics were those of 1989-90 (A/Shanghai/11/87) in the two countries [10].

The periods of the 2009 H1N1 pandemic were April 2009 to January 2010 in the US [11] and from September 2009 to January 2010 in France [12].

### Influenza data

We chose influenza-like illness (ILI) as the sole indicator of morbidity. The dates of the US seasonal epidemics and the age distribution of ILI were extracted from published studies [8,13]. The French data were obtained from the Sentinel system [12], created in 1984. The latter is a nationwide network of general practitioners who report, in real time, the number of medical visits for ILI.

The US ILI case definition consisted of fever and cough or sore throat. The French consisted of fever >39°C, aches, and sore throat. Influenza was not virologically confirmed in the French dataset, while it was virologically confirmed in the US for the H1N1 epidemic in 1978-79 and the H3N2 epidemic in 1989-90.

The age distributions of ILI during the 2009 pandemic in the US were collected from the Centers for Disease Control and Prevention [11,14]. In France, the age distribution of ILI during the pandemic was collected from the Sentinel system [12].

The age distributions of influenza-related mortality were obtained from national death registries: the National Bureau of Economic Research for the US [15] and the Centre d'épidémiologie des causes médicales de décès for France [16]. Influenza was identified using codes 470 to 474 of the International Classification of Diseases (ICD) 8th revision before 1979, and code 487 of the 9th revision thereafter.

The age distributions of influenza-related mortality during the 2009 H1N1 pandemic were collected from the Centers for Disease Control and Prevention (CDC) [17] in the US and from the French equivalent of the CDC (Institut de Veille Sanitaire) in France [18].

### Demographic data

We used yearly population data obtained from regular censuses. The age distribution of the national populations was obtained from the National Cancer Institute Surveillance Epidemiology and End Results for the US [19] and the Institut National de la Statistique et des Etudes Economiques for France [20].

The age distributions of all-cause deaths were obtained from the national death registries for seasonal epidemic periods [15,16]. As the 2009 data were not yet available at the time of the study, we used 2004/2005 mortality data - the most recent period in the WHO database [21].

### Ratios

We calculated age-standardized ratios (Relative Illness Ratio and Relative Mortality Ratio) in order to compare the age distribution of morbidity and mortality due to influenza between the two countries and between seasonal influenza epidemic and influenza pandemic. We first calculated the relative illness ratio (RIR), as the ratio of the percentage of sick persons in a given age group to the percentage of the general population belonging to the same age group. We then calculated the ratio between the percentage of influenza deaths in a given age group and the percentage of all-cause deaths in the same age group, yielding the relative mortality ratio (RMR). For both these ratios, a ratio above 1 indicates an excess risk.

The ratios were calculated as follows:

Relative Illness Ratio (RIR): (C_i_/Σ C_i_)/(N_i_/Σ N_i_)

C_i_: number of cases of influenza-like illness in a given age group

Σ C_i_: Sum of cases of influenza-like illness in all age groups

N_i_: Population in a given age group

Σ N_i_: Sum of populations in all age groups.

Relative Mortality Ratio (RMR): (I_i_/Σ I_i_)/(Di/Σ D_i_)

Ii: number of influenza deaths in a given age group

Σ I_i_: Sum of influenza deaths in all age groups

D_i_: Number of all-cause deaths in a given age group

Σ D_i_: Sum of all-cause deaths in all age groups.

### Statistical analysis

Confidence intervals for the RIR and RMR were calculated with an exact method based on the Poisson distribution [22]. Non parametric analysis of variance (Kruskall-Wallis test) was used to compare RIR and RMR values between epidemic and pandemic influenza. All statistical tests were two-tailed, with a type I error of 5%.

## Results

**Figure 1:**
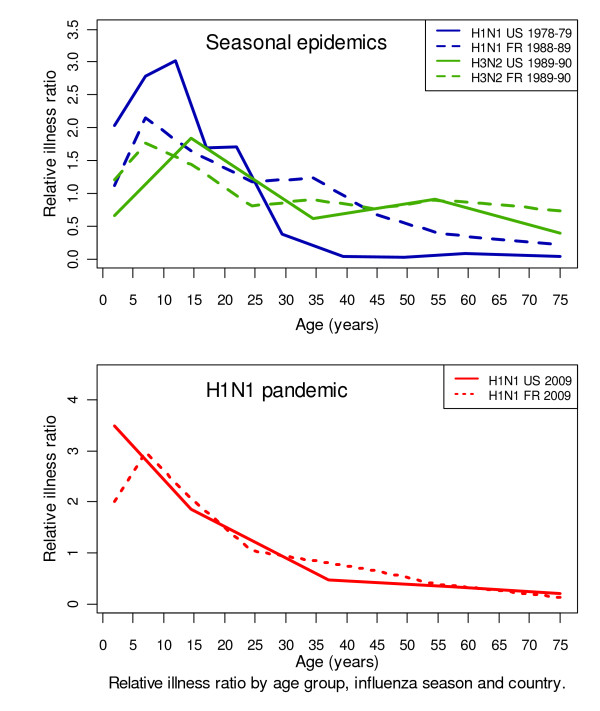
**Relative illness ratio (RIR) across age groups. The RIR is the ratio of the percentage of sick persons in a given age group to the percentage of the general population belonging to the same age group**. The age distribution of morbidity were established from data based on cohorts of 6,000 patients during the 1978-79 seasonal epidemic and 59,785 patients during the 1989-90 seasonal epidemic in the US. The age distribution of morbidity were obtained from the report of 18,839 (1988-89) and 27,054 (1989-90) cases in France. Pandemic age-distribution were obtained from 11,485 (France) and 658,078 (US) cases.

The RIRs are shown in figure 1 according to age. Overall, the 2009 H1N1 pandemic in the US and France resembled the seasonal epidemics of H1N1 (p = 0.72) and H3N2 influenza (p = 0.68).

Interestingly, the 2009 H1N1 pandemic was associated with RIRs above 1 among people under 35 years of age. Maximal RIR values of 2.97 (95% CI 1.76-4.71) and 3.49 (95% CI 2.23-5.20), respectively, were obtained for the 5- to 9-year age group in France and for the 0- to 4-year age group in the US. The maximal RIR values for the seasonal epidemics in France were obtained for the 5- to 9-year age group during the 1988-89 H1N1 epidemic (2.15 (95% CI 1.20-3.56)) and the 5- to 9-year age group during the 1989-90 H3N2 epidemic (1.77 (95% CI 0.92-3.09)). In the US, the maximal RIR values were for the 10- to 14-year age group during the 1978-79 H1N1 epidemic (2.99 (95% CI 1.92-4.43)) and the 5- to 24-year age group during the 1989-90 H3N2 epidemic (1.84 (95% CI 1.38-2.41)). All RIRs for people aged 35 years or more were below 1.

**Figure 2:**
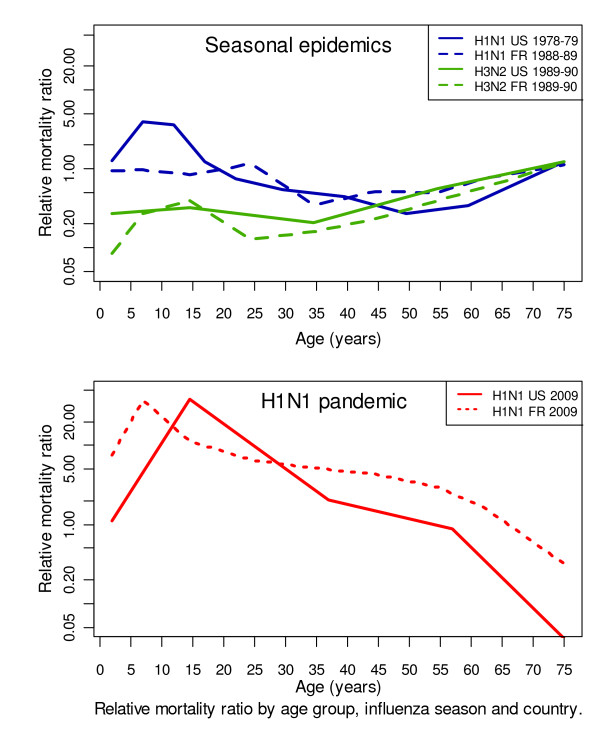
**Relative mortality ratio (RMR) across age groups. The RMR is the ratio between the percentage of influenza deaths in a given age group and the percentage of all-cause deaths in the same age group**. The age distribution of mortality due to influenza were established from the report of 368 influenza deaths during the 1978-79 seasonal epidemic and 1,926 influenza deaths during the 1989-90 seasonal epidemic in the US. The age distribution of mortality due to influenza were obtained from the report of 508 (1988-89) and 1,990 (1989-90) influenza deaths in France. Pandemic age-distribution were obtained from 308 (France) and 6,317 (US) influenza deaths.

Overall, the RMRs differed strongly between the 2009 H1N1 pandemic and the seasonal H1N1 (p = 0.007) and H3N2 epidemics (p = 0.0008) (figure 2).

RMRs above 1 were consistently observed among people under 60 years of age during the 2009 H1N1 pandemic waves, with maxima under 20 years of age. The maximal RMR values were 38.66 (95% CI 35.76-41.75) in the 5- to 24-year age group in the US and 37.39 (95% CI 24.40-54.83) in the 5- to 9-year age group in France.

Even though the RMR was above 1 for people under 60 years, the RMR fell significantly between the ages of 20 and 60 years (p = 0.04).

In contrast, during the seasonal epidemics, RMRs above 1 were consistently observed among people over 65 years of age. The RMR maxima in France were 1.12 (95% CI 1.04-1.20) during the 1988-89 H1N1 epidemic and 1.20 (95% CI 1.13-1.28) during the 1989-90 H3N2 epidemic; in the US, the maximal RMR value was 1.22 (95% CI 1.14-1.31) during the seasonal H3N2 epidemic. During the 1978-79 seasonal H1N1 epidemic in the US, the maximal RMR was in the 5- to 24-year age group (3.91 (95% CI 1.94-7.02)).

## Discussion

Although the age distribution of morbidity due to influenza was similar during the 2009 H1N1 pandemic waves and seasonal epidemics in the US and France, the age distribution of influenza mortality differed markedly. As reported ILI incidence rates for the 2009 H1N1 pandemic are unreliable, we chose to analyze the age distribution of ILI. For consistency, we used a similar method to analyze mortality data. However, it should be pointed out that the calculated relative illness and death ratios provide no information on the morbidity or mortality burden of influenza in the general population.

Our objective was to compare the age distributions of ILI between pandemic influenza and seasonal epidemics, and not to compare the burden of ILI (i.e. the cumulative incidence rates) between the US and France. The two data sources are not comparable, as some cases of ILI without virological confirmation could have been due to respiratory pathogens other than influenza virus. However, it is worth noting that the age distribution of these two sources of seasonal ILI (one virologically confirmed, the other not) were very similar, indicating no age-related bias in the dataset for ILI without virological confirmation and no difference between the risk of infection and risk of ILI. The use of ILI without virological confirmation tends to overestimate the ILI incidence rate but does not introduce a differential age-related bias if one assumes that the probability of a general practitioner making a wrong diagnosis is same in all age groups.

We cannot rule out the existence of a differential bias related to the use of different criteria for ILI and influenza-related death between the two countries, but the use of the age distribution of ILI and death would limit any such bias.

The seasonal epidemics chosen for comparison with the 2009 H1N1 pandemic date back between 20 and 30 years, and patient care no doubt evolved between 1978 and 2009, possibly impacting the incidence rates of morbidity and mortality. However, once again, we focused solely on the age distribution, which would be less affected by advances in patient care: the highest attack rate during seasonal epidemics is always observed among young people and the highest mortality rate among elderly people [23,24].

Although we studied only a few influenza seasons, the age distributions of ILI and death were very similar to those found in studies of several other influenza seasons [25,26]. Unlike the incidence of ILI and death, which can be highly variable from one influenza season to another (even with the same subtype), the age distribution of ILI and deaths is very similar across influenza seasons due to a given subtype [8,25].

Our calculated relative illness ratios showed no major differences between the 2009 H1N1 pandemic waves and seasonal influenza epidemics, suggesting that the age distribution of risk of infection did not differ. This might be explained either by similar age-to-age contact rates, or by preexisting immunity in older subjects [27]. Several studies of the age distribution of ILI during seasonal epidemics have shown that school-age children are most susceptible to contracting influenza [8,28]. Studies of the 1918, 1957 and 1968 pandemics showed an age distribution of morbidity similar to that of seasonal epidemics [29,30]. This is supported by a positive correlation between age and seroprotection from influenza [25]. One serological study showed that 30% of elderly people in the US had neutralizing antibodies to the pandemic H1N1 virus, attributed to exposure to H1N1 viruses that circulated until 1957, when H1N1 was replaced by a type A H2N2 virus [6,31].

In contrast, the relative mortality ratios differed between seasonal and 2009 H1N1 pandemic influenza. During the pandemic waves, these ratios were consistently above 1 among people under 60 years of age, peaking under the age of 20 years. This age distribution of mortality might be explained by lower preexisting immunity to the pandemic H1N1 virus than to seasonal viruses among the under-60s. However, we found that the RMR declined between the ages of 20 and 60 years. Prior exposure to seasonal influenza viruses thus seems to protect against the 2009 pH1N1 virus. This is supported by two studies, in which prior exposure to H1N1 seasonal type A influenza strains provided partial immunity against 2009 pH1N1 infection [32,33].

The US H1N1 epidemic in winter 1978-79 was due to a virus which re-emerged after a 20-year hiatus [8,28]. The results shown in figure 2 suggest that individuals aged over 20 years in 1978-79 were "protected" against this variant. We obtained similar results for persons aged over 60 years during the 2009 H1N1 pandemic. Although one study showed excess mortality due to the 2009 pH1N1 virus among the elderly [34], our findings are consistent with the results of several other studies [3,6,35,36]. Many elderly people would have had their immunity primed during childhood ("original antigenic sin" [37]) by a virus with similar antigenic properties.

## Conclusion

In conclusion, the age distribution of influenza-like illness was similar between the 2009 H1N1 pandemic and seasonal epidemics whereas the proportion of under-60s among influenza deaths was markedly higher during the 2009 pandemic (peak <20 years) than during the seasonal epidemics. However, a decrease of RMR between the age of 20 and 60 years was detected. This points to immunological cross-reactivity between the H1N1 influenza strain which circulated before 1957 and the 2009 pH1N1 strain, and also between seasonal influenza virus strains and the 2009 pH1N1 virus. These findings may have contributed to lowering the burden of the 2009 H1N1 pandemic but showed that young age was a principal mortality risk factor of the 2009 pandemic.

## Competing interests

F Carrat has received consulting fees from Roche, Aventis, Chiron-Novartis and attended sponsor-funded meetings.

M Lemaitre declares she has no potential conflicts of interest.

## Authors' contributions

FC and ML conceived the study, participated in its design and coordination, and drafted the manuscript. They both read and approved the final manuscript.

ML was responsible for data acquisition and statistical analysis.

## Author information

ML received her Master's in Epidemiology at Paris 11 University in June 2006. Since October 2006, she has been a PhD student in Epidemiology at UMR-S 707 ("Epidemiology, Information Systems, Modeling"). Her research focuses on influenza epidemiology, and particularly the role of immunity in the spread of influenza. FC is a Senior Lecturer in Epidemiology at Institut National de la Santé et de la Recherche Médicale (INSERM), Paris; Professor at the Department of Public Health, Université Pierre et Marie Curie, Paris; and Public Health practitioner at Saint-Antoine Hospital, Paris. He has been involved in influenza epidemiological research since 1992. His research interests include influenza epidemiology and modelling, surveillance, burden-of-illness, and evaluation of preventive interventions. He is also a member of a number of public health expert panels on influenza pandemic planning.

## Pre-publication history

The pre-publication history for this paper can be accessed here:

http://www.biomedcentral.com/1471-2334/10/162/prepub
